# High Epidermal Growth Factor Receptor Mutation Rates in Peruvian Patients With Non–Small-Cell Lung Cancer: Is It a Matter of Asian Ancestry?

**DOI:** 10.1200/JGO.2016.008201

**Published:** 2017-01-18

**Authors:** Joseph A. Pinto, Luis A. Mas, Henry L. Gomez

**Affiliations:** **Joseph A. Pinto**, **Luis A. Mas**, and **Henry L. Gomez** Oncosalud-AUNA, San Borja; **Luis A. Mas** and **Henry L. Gomez**, Instituto Nacional de Enfermedades Neoplasicas, Lima, Peru

In a recent article, Lopez-Chavez et al^1^ reported a high mutational rate of epidermal growth factor receptor (*EGFR*) in Peruvian patients (37%) that is higher than in other Latin American countries such as Mexico, Bolivia, Venezuela, and in a mixture of Latinos in the United States. High mutational rates of the *EGFR* gene in Peruvian patients were reported previously in independent cohorts. Mas et al^2^ reported a frequency of 39.3% (n = 122), and Arrieta et al^3^ reported a frequency of 51.1% (n = 393). Although the frequency of *EGFR* mutations in Peruvian patients is higher than other reports, these rates could be explained by environmental factors.

However, ancestry could also play an important role in explaining this fact. We would like to point out two events that could lead to a gene flow explaining the high prevalence of *EGFR* mutations in Peruvian patients. Population of the Americas in the late Pleistocene epoch by migrants from Asia through the Bering land bridge shaped the genetic pool of Native Americans. The second event occurred after slavery was abolished in Peru and a massive wave of Chinese workers reached the Peruvian coast (approximately 100,000 between 1849 and 1880), with a Peruvian population estimated at 2 million in 1850.^4,5^

**Fig 1:**
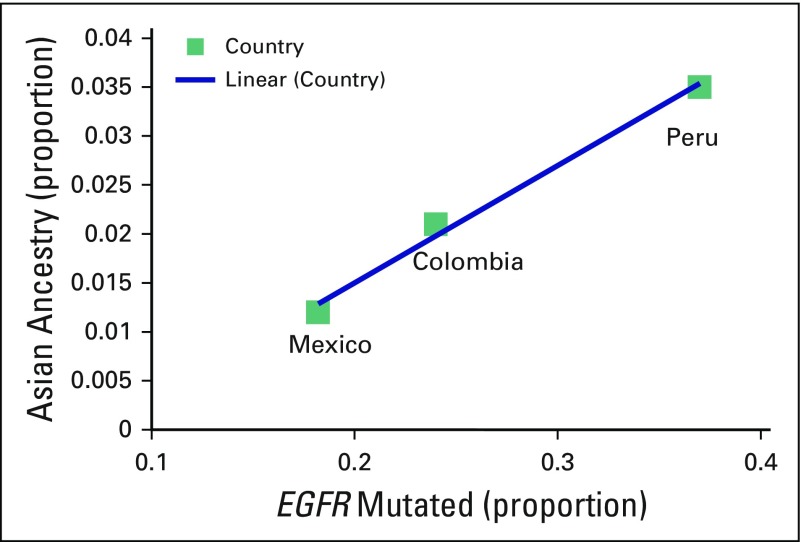
Graph showing a correlation between the proportion of Asian ancestry in Latin American countries and rates of *EGFR* mutations in non–small-cell lung cancer. *EGFR*, epidermal growth factor receptor.

Although there are not many projects that are evaluating Asian ancestry markers in Latin American countries, data for ancestry admixture proportions for Mexico, Colombia, and Peru (0.012, 0.021, and 0.035, respectively) suggest a correlation between ancestry proportion and rate of *EGFR* mutations (Fig 1).^1,6-8^

On the other hand, the *Helicobacter pylori* bacterium accompanied humans in the migration waves. These bacteria are not only a chronic pathogen in humans, but also coevolve with their hosts and have been used to trace human migration routes.^9^ Work by Devi et al^10^ with Peruvian strains of *H. pylori* found considerable homology with Asian strains. Another interesting fact is the high prevalence of human T-cell lymphotropic virus, ranging from 7% to 25%, in several Peruvian cities. This pattern is typical of some Asian countries such as Japan.

High rates of *EGFR* mutations in Peruvian patients with non–small-cell lung cancer could be a signature of Asian ancestry in the Peruvian population.
